# Pesticide Contamination in Native North American Crops, Part I—Development of a Baseline and Comparison of Honey Bee Exposure to Residues in Lowbush Blueberry and Cranberry

**DOI:** 10.3390/insects15070489

**Published:** 2024-06-29

**Authors:** Anne L. Averill, Brian D. Eitzer, Francis A. Drummond

**Affiliations:** 1 Department of Environmental Conservation, University of Massachusetts, Amherst, MA 01003, USA; averill@eco.umass.edu; 2 Department of Analytical Chemistry, The Connecticut Agricultural Experiment Station, New Haven, CT 06511, USA; brian.eitzer@ct.gov; 3 School of Biology and Ecology, University of Maine, Orono, ME 04469, USA; 4 Cooperative Extension, University of Maine, Orono, ME 04469, USA

**Keywords:** trapped pollen, pesticide application, risk quotient, pesticide class, field decay rates

## Abstract

**Simple Summary:**

Working in two native berry crops, we trapped honey bee pollen as foragers entered the hive. We then obtained grower records of the pesticides that were applied on the farm. We analyzed the pesticide residues in each pollen sample and established a baseline for real-world exposure levels and combinations to contaminated pollen. Between the two crops, the number of pesticides, total residue concentrations, and risk of exposure varied. The blueberry residue array was dominated by fungicides and miticides and cranberry was dominated by insecticides and herbicides. In most cases, pesticide residue concentrations were of low risk (low risk quotient) to honey bees in these crops. We documented that there were many residues that foragers would have picked up “off-farm.” The reports from growers regarding their pesticide application dates allowed estimates of field decay rates of several common pesticides. Some compounds were detected in pollen many days after application. Taken together, our findings may be key to future work aimed at reducing risk to bees.

**Abstract:**

A pesticide exposure baseline for honey bees was compiled for two New England cropping systems, the native North American plant species consisting of lowbush blueberry (*Vaccinium angustifolium* Aiton) and cranberry (*Vaccinium macrocarpon* Aiton). More unique pesticide compounds were applied in blueberry than cranberry, but the numbers of pesticides discovered in trapped honey bee pollen were similar between the two crop systems. Not all pesticides found in pollen were the result of the applications reported by growers of either crop. When comparing residues, number of pesticides detected, total concentration, and risk quotient varied between the two crops. Also, blueberry was dominated by fungicides and miticides (varroacides) and cranberry was dominated by insecticides and herbicides. When comparing reported grower applications that were matched with detection in residues, the proportion of pesticide numbers, concentrations, and risk quotients varied by crop system and pesticide class. In most cases, pesticide residue concentrations were of low risk (low risk quotient) to honey bees in these crops. Estimation of decay rates of some of the most common pesticide residues under field conditions could aid growers in selection of less persistent compounds, together with safe application dates, prior to bringing in honey bees for pollination.

## 1. Introduction

Honey bees (*Apis mellifera* L.) are critically important pollinators [1,2] and commercial beekeeping is vital, particularly for mass bloom produced by extensive monoculture plantings. However, beekeepers have been increasingly challenged by high losses, thought to be in part due to stress imposed by parasitic varroa mites [3], pathogen infection [4,5,6] and/or poor nutrition [7,8]. In combination with these stresses, exposure of honey bees foraging in pesticide-treated fields is often linked to increased probability of a colony’s morbidity or mortality [4,5,9,10,11].

Many lab and field studies have revealed that pesticide compounds alone may be toxic or have a constellation of sublethal consequences on performance, such as adversely affecting colony development or foraging behaviors [12,13]. In the past, the majority of studies emphasized the risk posed by contact exposure in sprayed fields. However, such actions fail to address compounds with systemic properties that can contaminate pollen and nectar. Neonicotinoid applications have come to dominate in many crops due to their systemic and high residual properties, low mammalian toxicity, and effectiveness [14]. However, even at exceedingly low levels, neonicotinoids can have lethal and sublethal effects on bees, and in many locations and crops, may be the greatest contributor to overall toxic load (number of lethal doses applied) to a foraging area [15,16].

Prior to the neonicotinoids becoming entrenched in many management programs, a noteworthy study evaluated the pesticide exposure of migratory honey bees and tracked colonies as they were moved among holding sites and crops. Operation Routes of the beekeepers followed east coast crops, including apple, blueberry, citrus, cucurbit, and cranberry. Residues from bees, stored pollen, and wax, together with hive condition, were recorded. When various measures of pesticide residue and hazard quotient (HQ) values [17] were compared among crops, pollination events of cranberry and blueberry were singled out as particularly deleterious in light of colony loss. For example, fungicide residues in samples from cranberry-associated bees were linked with imminent colony mortality as were HQ scores associated with fungicides in blueberry. Overall, they concluded that the cranberry environment is “a high-risk crop for honey bees” [5].

Here, we report follow-up studies of honey bee exposure risk in cranberry and lowbush blueberry. Cranberry production in southeastern Massachusetts and lowbush blueberry production in southern Maine occur in concentrated crop regions. Fungal pathogens causing fruit rot are often managed during cranberry bloom with broad-spectrum fungicides, particularly chlorothalonil. Key insect pests include fruitworms that are targeted during bloom. Both cranberry and blueberry are highly dependent on pollination services and for cranberry, only insecticides with a low-risk profile for bees are registered for use during bloom. Typically, no pesticides are applied during lowbush blueberry bloom, although immediately prior to bloom, fungicides and some insecticides are applied [18,19].

We sampled pollen collected by returning foragers when the hives had been deployed at blooming lowbush blueberry fields or cranberry bogs. We evaluated frequency of detections for different pesticide categories (active ingredients of insecticides, fungicides, herbicides, and acaracides). We compared the pesticide residues and acute risk quotients (RQ) in collected pollen. RQ values can account for the amount of pesticide consumed. Values can be compared to an established level of concern (LOC). For analysis of a residue, the ratio of daily exposure is divided by the acute oral LD_50_ and this evaluation has largely replaced utilization of HQ, which may overestimate risk [20,21,22,23].

Along with several beekeepers, we worked with grower cooperators that provided all of their pesticide applications and dates. This allowed a number of options: (1) to estimate daily decay rates of some of the most common pesticide residues under field conditions in each crop, (2) to determine frequency of detections from our residue analysis, (3) to establish whether there is a reliable link between a grower’s pesticide records and pesticide detection and concentration in trapped pollen and (4) determine whether non-crop, off-site pesticides were measurably contributing to the residues that we detected in bees stationed at the various farms. We also focused on miticides, assumed to be delivered into hives by beekeepers for the control of the parasitic Varroa mites, *Varroa destructor* (Anderson and Trueman) and *Varroa jacobsoni* Oudemans. In this study, we assumed that residues of amitraz, coumaphos, and fluvalinate or their metabolites detected in trapped honey bee pollen were applied to colonies in hives by beekeepers for control of Varroa mites as these compounds are not used in blueberry or cranberry insect pest management in the northeastern U.S. [24,25].

## 2. Materials and Methods 

### 2.1. Maine Lowbush Blueberry Sites

Sampling of pesticide residues associated with lowbush blueberry fields during bloom was conducted in the two major lowbush blueberry growing regions in Maine, USA. Blueberry fields were selected in a two-stage process. First, we contacted commercial lowbush blueberry growers to determine if they would be willing to cooperate with our research project. These growers were located in the two major blueberry growing regions (Midcoast and Downeast) in the following Maine counties: Hancock, Penobscot, Sagadahoc, Waldo, and Washington. The growers initially contacted were either conventional or organic producers who used standard practices recommended by the University of Maine Cooperative Extension Service for crop management. Second, we contacted the beekeepers who deployed hives on the potential cooperating growers’ fields to determine if they would be willing to cooperate. The growers’ fields, different fields in each of three years, were then selected in a manner that provided a representative geographic and management intensity sample of growers’ fields throughout the two growing regions. Fields are typically surrounded by a mixed deciduous conifer forest in the Midcoast region and a spruce-fir forest in the Downeast region. This is because lowbush blueberries are an understory plant species and field are created by clearcutting the forest in which they reside. Fields are typically managed on a two-year production cycle where every other year is characterized by bloom and fruit harvest. All sampled fields in each year were a minimum of 3.1 km from one another, greater than the 1 km average foraging distance [26] and greater than 2.0 km, the average maximum foraging distance of honey bees [27]. Field size ranged from 1 to 72.8 ha with a mean of 16.8 ± 2.4 (s.e.) ha.

#### 2.1.1. Sample Collection

Twelve fields were sampled in 2012, 13 fields in 2013, and 14 fields were sampled in 2014. Of these fields, four individual fields were repeatedly sampled annually for a total of 36 unique fields that were sampled. Thirty of the blueberry fields were managed with conventional pest management and fertility tactics while six of the fields were managed with organic tactics [28,29,30,31,32]. All sampling was conducted during full bloom [(20–26 May 2012), (31 May–6 June 2013), and (25 May–12 June 2014)].

Sampling to determine levels of pesticide exposure to honey bees was performed by trapping pollen at honey bee hive entrances. In each of the 36 fields, migratory honey bee hives were placed by beekeepers at the start of bloom on wooden pallets with 4–6 hives per pallet. The numbers of hives per field ranged from 4 to 180, depending upon field size and stocking density (range: 2.5–20 hives/ha).

At each field in each year, three random hives were selected from the total aggregation in a field. Pollen was collected from foraging honey bees returning to the hive during peak bloom using a front entrance pollen trap (Betterbee, Greenwich, NY, USA). This was performed once each year. Pollen collected from all three pollen traps deployed in a field was pooled.

#### 2.1.2. Storage of Samples

All residue samples were transported at the end of the day of collection from the field to the laboratory in Orono, ME in insulated coolers (The Coleman Co., Inc., Golden, CO, USA) containing blue ice packs (Igloo Maxcold^®^ ice blocks, Igloo Co., Katy, TX, USA). Once at the laboratory, samples were transferred to labeled 100 mL plastic vials.

#### 2.1.3. Grower Pesticide Application Records

Copies of pesticide application records were received from all of the cooperating growers. Only the pesticides applied before and during bloom in each year were used. It is common for lowbush blueberry growers to split their fields in two sections and manage one section for vegetative growth after pruning and one section for fruit production. Each adjacent section is managed on an alternate vegetative/fruit production 2-year cycle. Where this was the case, the pesticide application records were requested for both field sections, fruit bearing and pruned for each year of this study.

### 2.2. Massachusetts Cranberry Sites

Samples for pesticide analysis were collected at commercial blooming cranberry farms in the New England coastal zone of Massachusetts, USA. Cranberry beds were picked so that there would be representation across all cranberry-growing counties (Plymouth, Bristol, Barnstable). We selected effective cooperators where bees were installed and where conventional practices were applied. All sites were located in landscapes characterized by sandy and acidic soils that support *Pinus rigida* Mill and *Quercus ilicifolia* Wangenh forests. Sites were situated within the region’s sand plains in two sub-ecoregions, Bristol Lowlands and Cape Cod and the Islands. The sites were surrounded predominantly by residential communities except for MA-6, which was situated in Myles Standish State Forest, Carver, MA, USA. There is no other significant agriculture in the area. Sites MA-6 and MA-8 belong to the University of MA and are experimental farms, but are managed conventionally. An overview of current management methods for cranberry is found in Ghantous et al. (2024) [33] and historical management methods at the time of the study are found in Sylvia and Gauvin (2012) [24]. Beds adjacent to collection sites ranged in size from 3.2 to 70 ha, with an average area of 11.1 ha.

#### 2.2.1. Sample Collection

Pollen trapping from honey bee hives was performed for cranberry in 2012 and 2013. Migratory beekeeping operations installed rental hives at all but three sites, where stationary hives were maintained by a single local beekeeper. In 2012, two migratory beekeepers and in 2013, five different beekeepers provided the rental hives. At peak cranberry bloom (mid-June to mid-July), we utilized the same front-mounted pollen traps (Betterbee, Greenwich, NY) as in blueberry. To determine the relationship between pesticide applications to beds and the corresponding residues found in cranberry pollen samples, we sampled pollen at 10 farms in 2012 and nine farms in 2013. We expanded the number of sites when we sought to establish residue classes in pollen, including acaracides, and collected pollen from 13 sites in 2012 and from 20 sites in 2013. Pollen was trapped a single time at all sites with the exception of multiple trapping events 7–10 days apart at MA-16 (two events in 2012), MA-3 (two events in 2013), MA-8 (3 events in 2013) and MA-23 (2 events in 2013).

#### 2.2.2. Grower Pesticide Application Records

At the end of each of the growing seasons, we obtained copies of pesticide application records from cooperating growers. Only the pesticides applied before and during bloom were used.

### 2.3. Storing, Shipping, and Analytical Methods Applied for Sample Handling and Residue Analysis

All samples were stored at −80 °C in ultra-freezers (Thermo Scientific^®^, Fisher Scientific, Hampton, NH, USA). Samples were shipped overnight express on dry ice to the quantitative chemical analytical laboratory at the Connecticut Agricultural Experiment Station, New Haven, CT, USA.

Samples were analyzed using a modified Quechers extraction protocol [17]. In brief, pollen samples for each site and year (5 g if sufficient pollen was available or entire sample if less) were combined with water to a final volume of 15 mL. To each sample was added 100 ng of isotopically labeled (d-4) imidacloprid (Cambridge Isotope Laboratories Andover, MA, USA) as an internal standard. Although this does some correction for recovery, it would not account for matrix effects on each individual pesticide (which was cost prohibitive). The samples were combined with 15 mL of acetonitrile, 6 g magnesium sulfate and 1.5 g sodium acetate, and 150 uL of acetic acid. After shaking and centrifuging, 10 mL of the supernatant was combined with 1.5 g magnesium sulfate, 0.5 g PSA, 0.5 g C-18 silica and 2 mL toluene. The samples were further shaken and centrifuged. Finally, 6 mL of the supernatant was concentrated to 1 mL for instrumental analysis.

Pollen residues were analyzed with liquid chromatography/mass spectrometry/mass spectrometry (LC/MS/MS). The LC system was an Agilent 1200 Rapid Resolution system with a Zorbax SB-C18 (Agilent, Santa Clara, CA, USA) Rapid Resolution HT 2.1 × 50 mm, 1.8 μm column using a 3 μL injection with the gradient going from 5% methanol in water to 100% methanol at 0.45 mL/min. In both cases the LC was coupled to a Thermo-LTQ (Thermo-Fisher, Waltham, MA, USA, a linear ion trap mass spectrometer. The system was operated in the positive ion electrospray mode, with a unique scan function for each compound allowing for MS/MS monitoring. Detection limits varied with the amount of sample available but were in the single ng pesticide/g pollen level [commonly abbreviated as ppb (parts per billion)] or lower for pesticides reported herein. If a pesticide was not detected, it was reported as such, not as a concentration of 0. Though our procedures were similar to those reported by Mullin et al. [34], we do not report on the same set of pesticides as does that study. For enhanced specificity in pesticide detection at very low concentrations, we chose to use only our available LC/MS–MS. While providing greater confidence in our data on pesticides detected during a multi-residue screen, this choice precluded detection of some classes of pesticides (such as pyrethroid insecticides; also we could not detect chlorothalonil, only its hydroxyl metabolite), which can require additional sample cleanup steps and GC/MS in electron impact or negative chemical ionization modes.

### 2.4. Statistical Methods

All statistical analyses were conducted in JMP PRO version 16 [35]. Pesticide residues in both of the cropping systems were ranked and a list of the top 10 most frequently occurring residues was compiled along with their concentrations in trapped honey bee pollen: MA (2012–2013) and ME (2012–2014). Risk quotients (ratio of daily exposure through pesticide consumption divided by acute oral LD_50_) were calculated from the detected compound-specific pesticide or pesticide degradation concentrations, oral LD_50_s, and honey bee adult consumption rates based upon the methods cited in Hester et al. (2023) [23] and the US-EPA BeeREX tool [36]. In a few cases, the oral LD_50_ estimate was not available and so the contact LD_50_ value had to be substituted for the oral LD_50_ value. The LD_50s_ for each specific pesticide and/or metabolite residue were based upon values published by Ostiguy et al. (2019) [37] and the web-based database ECOTOX [38]. A daily adult honey bee pollen consumption rate of 9.5 mg/day was utilized [13]. An acute RQ > 0.4 exceeds the concern threshold and suggests a high toxicity [36].

Kendall’s tau monotonic correlation coefficient was used to estimate the association of the frequency and concentration of shared pesticide residues within and between cropping systems (Kendall’s tau monotonic correlation coefficient was estimated for shared residues among cropping systems) [35]. Kendall’s tau was selected because it does not assume a linear relationship between variables and it is robust to small sample sizes [39].

Grower’s pesticide application records were used to determine the number of pesticides applied per field per year in each crop system and the proportion of residues that were likely a result of an application compared to those residues that were likely due to exposure outside of the growers’ fields. Using the grower pesticide application records and the list of pesticide detections in honey bee trapped pollen, variables were calculated that estimated: (1) the proportion of residues detected but not sprayed by a grower at a specific farm: the number of residues detected that were not sprayed on a farm (based upon grower application records)/total number of pesticide residues detected on a farm; (2) the proportion of a grower’s sprays detected on a farm: spray residues detected (based upon grower application records)/the number of pesticides applied by the grower; (3) the proportion of concentration of pesticide residues that a grower applied on the farm: total concentration (ppb) of pesticide residues that a grower sprayed (based upon grower application records)/total concentration (ppb) of pesticide residues detected in trapped pollen at the grower’s farm, and (4) the proportion of risk quotient due to a grower’s sprays: sum of risk quotients of each pesticide residue that grower sprayed/sum of risk quotients from the total residue concentrations detected in the trapped pollen on a farm.

In addition to the total number of pesticides applied by growers, patterns of specific pesticide use classes were investigated. Measures we used to assess the impact of varroacides in trapped pollen were: (1) the proportion of varroacide compounds relative to the total residue numbers detected in trapped pollen, (2) the proportion of concentration of varroacides in trapped pollen from a farm: concentration (ppb) of varroacide in trapped pollen from a farm relative to the total residue concentration (ppb) detected in trapped pollen from a farm, and (3) the proportion of varroacide risk quotient in trapped pollen from a farm: sum of varroacide residue risk quotient/total summed risk quotient from all the pesticide residues detected in trapped pollen on a farm.

Grower-applied specific pesticide use classes that we investigated were fungicides, herbicides, and insecticides. The number of grower applications for each pesticide use class was derived from grower pesticide application records. We assumed that the detected residues in trapped pollen that matched the same specific pesticides that growers applied were the result of the applications and that pesticide residues that did not match pesticides applied by growers were from outside of the field/bog that hives were located in. Based upon these assumptions, we constructed the following metrics to assess the impact of grower-applied specific pesticide use classes. The metrics were: (1) the proportion of applied pesticide use class compounds relative to the total residue numbers detected in trapped pollen, (2) the proportion of applied pesticide use class compound concentrations in trapped pollen from a farm: concentration (ppb) of pesticide use class compounds in trapped pollen from a farm relative to the total residue concentration (ppb) detected in trapped pollen from a farm; and (3) the proportion of pesticide use class compound risk quotient in trapped pollen from a farm: sum of pesticide use class residue risk quotient/total summed risk quotient from all the pesticide residues detected in trapped pollen on a farm.

Mixed models were used to account for variation in fields, years, and sample periods within fields during bloom. Dependent variables were checked for normality by inspecting Normal quantile plots, assessing goodness of fit with the Shapiro–Wilk and Anderson-Darling tests [40] or failure of convergence in maximum likelihood estimation of linear mixed models. Model fit and appropriateness were evaluated with the χ^2^ convergence test. Fixed effects were tested for significance using Satterthwaite’s method for estimating denominator degrees of freedom in fixed effects F-tests in a mixed model using JMP [41]. Tukey HSD post hoc tests were used to determine mean separation of fixed effects with controlling Type I error [42]. Depending upon the model, dependent variables were: field size, the number of pesticide residue chemicals, residue concentrations measured in parts per billion (ppb), and risk quotient calculated with oral adult honey bee LD_50s_ for specific pesticides and metabolites and an adult honey bee pollen consumption rate of 9.5 mg/day (see [13] and previous methods for more detail). Fixed effects were cropping system, field size, and their interactions. Random effects were field, year, and sampling period (during early, mid, or late bloom) nested within field. For the pesticide residue group comparisons within each crop, the dependent variables were square root of the number of pesticide residues, log PPB or log RQ. Without these transformations either the maximum likelihood estimations would not converge or the model residuals were not found to be distributed in a Gaussian manner based upon quantile plots. In addition, mixed model repeated measures analysis was based upon a random effect measure of year and a random within-subject of field ID. The variance–covariance structure was modeled by first assessing the variance–covariance structure using a JMP add-in [43]. Based upon the results one of several appropriate variance–covariance structures were selected for the mixed model. In our case, the repeated covariance structures employed were either the Ante-dependent Unequal Variance structure or the Toeplitz Unequal Variance structure [43].

Grower pesticide application records were used to link pesticide residue concentration in trapped honey bee pollen to the number of days after the pesticide application in the field until the pollen was collected. Pesticide residues chosen for modeling were those that ranked high in frequency among growers and ranked high in concentration among all pesticide residues detected in trapped pollen. Poisson generalized regression [35] was used to develop models for the prediction of residue concentration. Predictors initially selected were year, days after application, and the interaction of year x days after application. Predictive variable selection was based upon the adaptive lasso technique [44] and the Poisson model coefficients were estimated with maximum likelihood. A generalized regression coefficient of determination (*r*^2^) was calculated as the Nagelkerke likelihood *r*^2^ [45]. Regression models that appeared to be highly leveraged by a single data point were run with and without the leveraging data point to determine the significance of the leverage. Leverage was indicated for those models that changed significantly with the deletion of the leveraging data point. Half-lives of residues (or the 50th percentiles of concentration residence time, abbreviated as RT_50_) on field-trapped pollen were estimated using the fit Poisson generalized regression models. Published ranges of RT_50_ values and the average RT_50_ estimates were obtained from the IUPAC Pesticide Properties Database (PPDB) found at: http://sitem.herts/ac/uk/aeru/iupac/atoz.htm (accessed on 10 June 2024). We compared our estimates of RT_50_ with the range of published RT_50_ values for each pesticide that was fit to a Poisson generalized regression model. If our model RT_50_ estimate was within the minimum and maximum range of published RT_50_ values, we concluded that our model estimate was in agreement with the published RT_50_.

## 3. Results

### 3.1. Pesticide Use and Honey Bee Exposure in Blueberry and Cranberry Farms

#### 3.1.1. The Number of Different Pesticides Applied by Growers in Blueberry and Cranberry Crops

**Figure 1:**
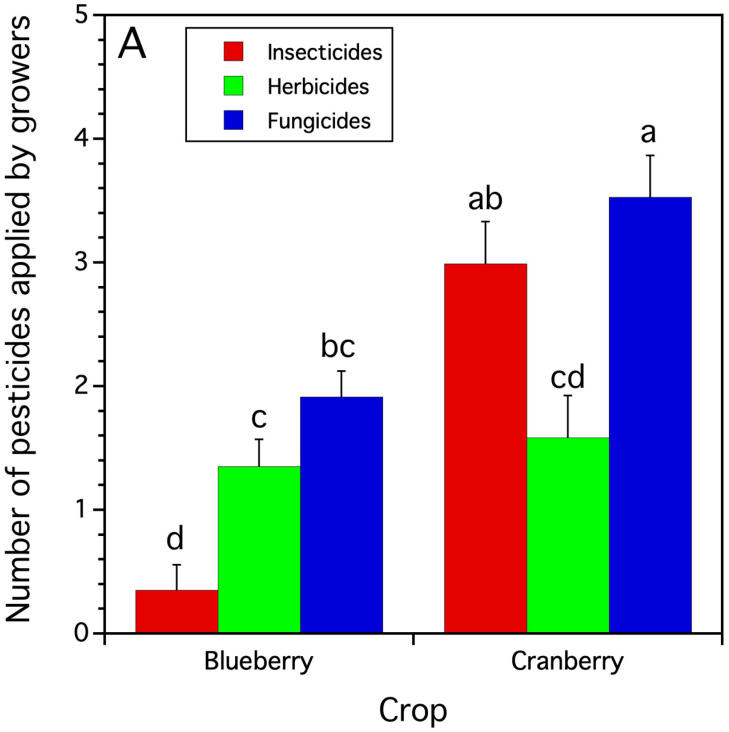
Average number/field or bog of grower-applied pesticides by class. Bars of the same residue measure followed by the same letters across crops and pesticide class are not significantly different from one another (Tukey HSD test, α = 0.05).

Grower application records showed that blueberry growers applied significantly fewer different pesticides in the spring than cranberry growers (*F*_(1,42.8)_ = 10.045, *p* = 0.003, blueberry = 3.7 ± 0.4 (s.e.) pesticides applied/field vs. cranberry = 6.3 ± 0.7 pesticides applied/bog). Blueberry and cranberry growers also applied pesticides differently according to pesticide class (crop x pesticide class interaction: *F*_(2,116.3)_ = 13.818, *p* < 0.0001). Figure 1 shows that blueberry growers applied equal numbers of fungicide and herbicide compounds and fewer insecticide compounds, while cranberry growers applied equal numbers of insecticides and fungicides and significantly fewer herbicides.

**Figure 2:**
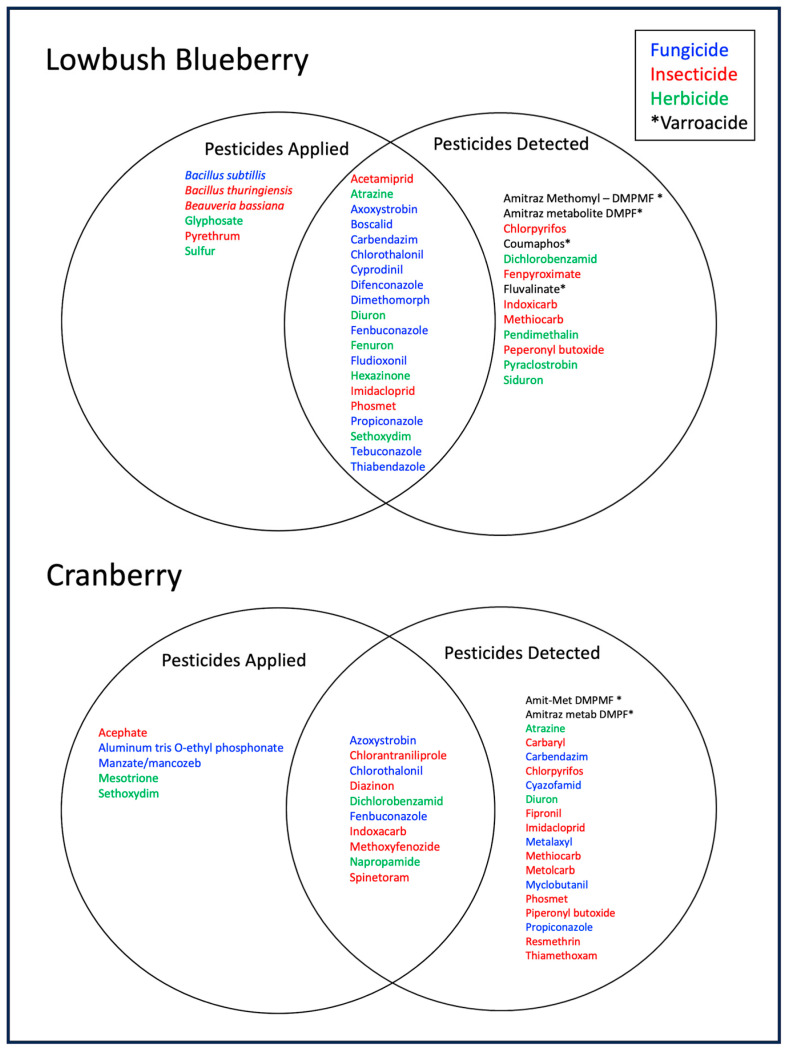
Two-set Venn diagrams for blueberry (**top**) and cranberry (**bottom**). Each Venn diagram is comprised of a circle or set of pesticides that growers applied in the study fields or bogs and a circle or set of those pesticide residues that were detected in trapped pollen in the same fields. The overlap or intersection of the two sets represents those pesticides that were applied by the growers and detected in trapped pollen. Pesticides detected that are not within the Pesticides applied set are those that were not applied by the growers on their fields or bogs, but were nonetheless detected in trapped pollen. Pesticide groups are designated by color where; insecticides are in red print, fungicides in blue print, herbicides in green print, and assumed *Varroa* mite targeted miticides in black print and followed by an asterisk.

Two-set Venn diagrams (Figure 2) delineate the pesticides that were applied by growers (left circles) on the study fields (blueberry) and bogs (cranberry). A further breakdown (right circles) shows pesticide residues that were detected in trapped pollen from those same fields or bogs. The intersection of the two circles (pesticides applied and pesticide residues detected in trapped pollen) in each crop system represents those pesticide residues detected in trapped pollen that were applied by the growers. Inspection of the two-set Venn diagrams across the two crop systems shows that there were more individual pesticide products applied by blueberry growers (*n* = 26) than cranberry growers (*n* = 15). There was little overlap in specific pesticides that were applied by growers among the two crops (Figure 2). The herbicide, sethoxydim, and the fungicides, azoxystrobin, chlorothalonil, and fenbuconazole, were applied in both cropping systems.

#### 3.1.2. Pesticide Residues Detected in Trapped Pollen in Blueberry and Cranberry Crops

The number of pesticides or their metabolites detected in trapped honey bee pollen was similar in number for blueberry (*n* = 33) and cranberry (*n* = 29) (Figure 2). The number of pesticides applied by growers and subsequently detected in trapped pollen were 20 of 26 or 76.9% for blueberry and 10 of 15 or 66.7% for cranberry. Thirteen pesticides that were not applied by blueberry growers were detected in trapped pollen from hives in the blueberry fields under the grower management (Figure 2). Nineteen pesticides that were not applied by growers were detected in trapped pollen from hives in their managed cranberry bogs (Figure 2).

A comparison of the most frequently detected pesticide residues in trapped honey bee pollen in each of the crop systems is presented in Table 1. The fungicide residues propiconazole in blueberry and 4-hydroxy-chlorothalonil in cranberry were found in 82 and 90% of all colonies sampled, respectively. In addition, it was common for trapped pollen samples in both blueberry and cranberry to have high prevalence (63–72%) of residues of the varroacide amitraz metabolite, Amit-Met DMPMF methomyl coelutes. Beekeepers use amitraz to reduce populations of *Varroa* mite. Blueberry had very high concentrations of amitraz residues compared to cranberry.

One difference between trapped pollen from blueberry and cranberry was that four insecticide residues were found in at least 25% of colonies sampled in cranberry, compared to only one insecticide being a top-ten residue in blueberry. Common to pollen trapped in the two cropping systems was the number of different fungicides (3–6) and the few herbicides (1–3) that occurred in the top-ten ranking residues. Only three specific residue compounds were common to the two cropping systems: the fungicide, 4-hydroxychlorothalonil and the two amitraz metabolites: Amit-Met DMPF, and Amit-Met DMPMF (methomyl coelutes). Overall, there were no significant Kendall rank correlations (*p* > 0.05) between the combined crop systems top-ten most frequently detected pesticide residue frequency of detection and concentration (ppb). There was also no significant rank correlation (*p* > 0.05) within each crop system between the frequency of detection of a residue and its concentration (ppb). Therefore, the most frequently detected pesticides were not the pesticides in the highest concentrations, although there was a non-significant trend for the Kendall rank correlation between cranberry frequency of detection and concentration (*r* = +0.449, *p* = 0.073).

### 3.2. Total Residue Pesticide Concentrations and Risk of Honey Bees to Provisioned Pollen

#### 3.2.1. Concentrations and Risk from Total Residues Detected in Trapped Pollen

**Figure 3:**
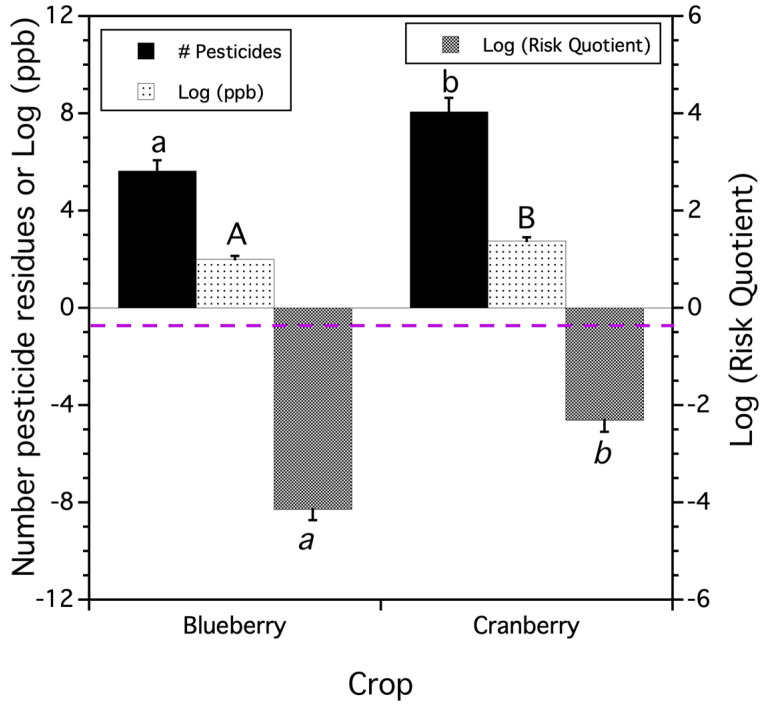
The mean number of pesticide residues per bog or field per year, mean logarithm transformed concentration of residues [log(ppb)], and mean logarithm risk quotient [log (RQ)] found in trapped honey bee pollen by crop. Means and standard errors are least square estimates from mixed models. Bars of the same residue measure with the same letters are not significantly different from one another (Tukey HSD test, α = 0.05). Dashed purple line is the EPA warning level for high toxicity. The more negative the mean logarithm RQ values, the less risk trapped pollen has to honey bee colonies.

There were significant differences in the mean number of pesticide compounds in samples of honey bee pollen trapped from colonies brought in for pollination of blueberry and cranberry (*F*_(1,43.6)_ = 11.219, *p* = 0.002). There was a higher number of pesticide and metabolite compounds detected in trapped cranberry pollen per bog per year than in blueberry pollen per field per year (Figure 3). The mean concentration of total or cumulative pesticide residue (log ppb) exhibited the same pattern (*F*_(1,29.4)_ = 14.358, *p* = 0.0007), with pollen trapped from colonies in cranberry bogs per year having higher pesticide residue concentrations than pollen trapped in blueberry fields per year (Figure 3). The total or cumulative risk quotient (RQ) from the concentrations of residues in trapped pollen also differed by crop system (*F*_(1,44.2)_ = 29.707, *p* < 0.0001). The log-transformed RQ was higher in pollen trapped in cranberry bogs per year compared to the log-transformed RQ in blueberry fields per year (Figure 3).

#### 3.2.2. Pesticide Use Class Total Residues

**Figure 4:**
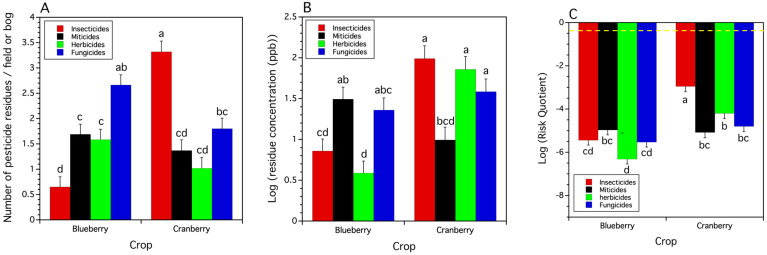
The number of pesticide compounds (**A**), pesticide use class concentrations of residues (log ppb) (**B**), and risk quotient of pesticide residues (**C**) in pesticide use classes (insecticides, miticides, herbicides, and fungicides for both crops. Pesticide use class bars with the same letters are not significantly different from one another (Tukey HSD test, α = 0.05). Means and standard errors are least square estimates from mixed models. Dashed yellow line in (**C**) is the EPA warning level for high toxicity. Lower risk quotient values suggest less risk to honey bee colonies.

The number of different classes of pesticides or their metabolites detected in trapped pollen differed both within and between blueberry and cranberry (*F*_(6,192.2)_ = 23.082, *p* < 0.0001). The average number of insecticides per bog found in cranberry trapped pollen was significantly higher (ca. five times) compared to blueberry trapped pollen per field (Figure 4A). All of the other pesticide use-classes showed no difference in the number of pesticide types between the two crops (Figure 4A). Within a crop, in blueberry, the number of insecticides/field was the lowest of the pesticide classes and fungicide numbers were highest.

The concentrations (log ppb) of pesticide use classes differed among pollen trapped both within and between the two crops. This was supported by a significant interaction between crop and pesticide use class (*F*_(6,170.3)_ = 11.012, *p* < 0.0001). Insecticides and herbicides appeared in a significantly higher concentration in cranberry compared to blueberry, while miticide and fungicide concentrations [log (ppb)] were similar for both (Figure 4B). Blueberry samples were dominated by concentrations of miticides and fungicides.

The RQs of the major groups of pesticides differed among the two crops and differed between pesticide use classes within crops. There was a significant interaction between crop and pesticide use class (*F*_(6,190.7)_ = 12.131, *p* < 0.0001). The insecticide use class had the highest RQ (log RQ) in cranberry trapped pollen and was significantly higher than the insecticide RQ of trapped pollen collected in blueberry fields (Figure 4C). Cranberry trapped pollen had a significantly higher mean herbicide RQ than pollen collected from blueberry fields (Figure 4C). The mean fungicide and mean miticide RQs were not different among the two crops (Figure 4C).

Within crop comparisons of mean RQs show that miticides, insecticides, and fungicides in trapped pollen collected in blueberry fields are not different from one another, but their mean RQs are all significantly higher than the RQ of herbicides (Figure 4C). Cranberry shows a different pattern. Trapped pollen had a significantly higher insecticide mean RQ than the RQs of miticides, fungicides, and herbicides, which were not different from each other (Figure 4C).

**Figure A1:**
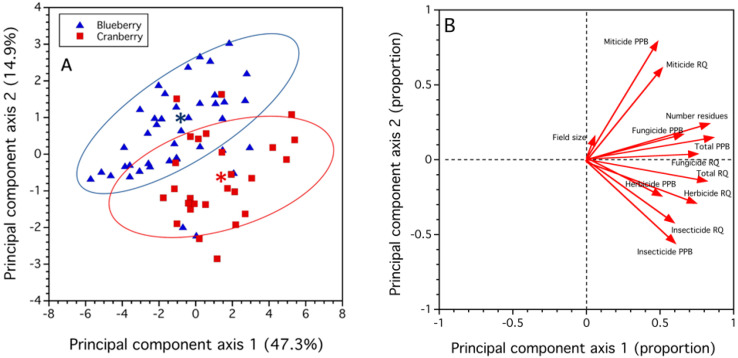
A principal component ordination constructed from the variables: field size, # pesticide residues, log (total ppb), log (total risk quotient), log (insecticide ppb), log (insecticide RQ), log (miticide ppb), log (miticide RQ), log (herbicide ppb), log (herbicide RQ), log (fungicide ppb), and log (fungicide RQ) (**A**). Blueberry field samples are depicted in blue triangles with the centroid of the data swarm depicted by a blue asterisk and Cranberry bog samples are depicted by red squares with the centroid depicted by a red asterisk. A biplot of the orthogonal factor loadings comprising the first two principal components shows the strength (length of arrow) and direction (arrows starting at the origin (0, 0) and directed toward two-dimensional coordinates in quadrats of the ordination space) (**B**).

The several significant differences in total pesticide residue concentration and risk quotient between crops just described are also the case when all pesticide class residue concentrations and risk quotients were investigated in a multi-dimensional Principal Component Analysis. However, there was also overlap between the data clouds among crops (Appendix A, Figure A1).

#### 3.2.3. Varroacide Residues in Trapped Pollen

One source of pesticide residue in honey bee trapped pollen that was not due to grower applications was the presumed varroacide (miticide) application by beekeepers. The proportion of the number of varroacides detected relative to the number of total pesticide residues detected were determined by crop (Table 2), but was not influenced by field or bog size (*p* = 0.21). In cranberry, the proportion of the number of varroacides detected compared to the total pesticide residues detected in pollen was an order of magnitude less than what was found in trapped pollen collected in blueberry fields (Table 2). The proportion of the concentration of varroacide residues (ppb) relative to the total residue concentrations (ppb) was also determined by crop (Table 2), but not influenced by field or bog size (*p* = 0.37). In cranberry, the proportion of the number of varroacides detected compared to the total pesticide residues detected in trapped pollen from cranberry bogs was less than in pollen originating from blueberry fields (Table 2). (76.9% of blueberry locations had detectable varroacide concentrations in trapped pollen, but only 47.4% of cranberry locations had trapped pollen with detectable varroacide concentrations). Despite this difference, the proportion of RQ derived from varroacide treatments relative to the total RQ found in trapped pollen did not differ among crops (Table 2), and as with all of the varroacide measures, field or bog size did not influence the proportion of varroacide RQ (*p* = 0.154).

### 3.3. Proportion of Total Residues in Trapped Pollen That Matched Grower Application Compounds

#### 3.3.1. Residues That Matched Pesticides Applied by Growers, a Proportion of Total Residues Detected in Trapped Pollen

Figure 2 lists the pesticide residues that were most likely a result of grower applications and those that were likely not a result of grower applications (residue compounds not matched by grower applications). The proportion of pesticides that was applied and also detected relative to the total number of residues detected in trapped pollen were similar for both crop systems (Table 2). This mixed model used to test the proportion of pesticide residues that matched application by growers also included a covariate fixed effect of field/bog size along with a crop systems x field size interaction. The interaction was not significant (*p* = 0.26). The main effect of field/bog size (ha) was significant, but only at the α = 0.10 level (*F*_(1,44.3)_ = 3.016, *p* = 0.057, β = 0.005). However, this was a weak effect across both cropping systems given the test probability value and a partial marginal coefficient of determination of 8.7%.

However, the proportion of pesticides that was applied and also detected relative to the total number of pesticides applied by the growers was different by crop system (Table 2). Blueberry demonstrated a significantly higher detection rate of applications (proportion of applications detected relative to total residues detected) than cranberry (Table 2). The trend of a decreasing detection of applications from blueberry, an early spring blooming crop, to cranberry, a late spring and summer blooming crop, may reflect the ambient temperatures during applications within each crop system with application dates becoming later from early spring to summer, increasing air temperatures and decreasing latitude as one transitions from the Maine blueberry to Massachusetts cranberry. However, this could also be due to the type and formulation of the pesticide applied that might have affected our ability to recover detectable residues.

#### 3.3.2. Concentrations and Risk Quotients of Grower Applications Relative to Total Residues

The proportion of the concentration (ppb) of residues in honey bee trapped pollen that growers presumably applied (based on grower application records) relative to the total pesticide residue concentration detected in trapped pollen varied by crop system (Table 2). Cranberry had a significantly higher proportion of residue concentration in trapped pollen attributed to grower application than what was observed in blueberry. Field or bog size had no influence on residue concentrations (*p* = 0.116). However, the proportion of RQ from residues in trapped pollen that was due to grower applications relative to the total RQ of residues detected was not significantly different by crop system. However, the RQ were highly variable from farm to farm and among years (Table 2). Field or bog size did influence RQ in trapped pollen collected from hives in specific locations (*F*_(1,38.4)_ = 4.822, *p* = 0.034). The interaction between crop and field or bog size was not significant (*p* = 0.892). The slope coefficient for field or bog size (β) was 0.008 ± 0.003, suggesting that larger fields and bogs had higher risk to honey bees, suggesting use of more toxic pesticides, higher doses or more frequent applications in the larger fields or bogs.

#### 3.3.3. Residues Matched to Grower Applications—Pesticide Use Classes

When the number of different compounds applied within each pesticide class (fungicide, herbicide, and insecticide) was compared between the two crops, a mixed model suggested an interaction between pesticide class and crop (Table 3). In cranberry, there were more fungicide and insecticide applications than herbicide applications. In blueberry, the number of fungicide applications did not differ from herbicide applications, and there were fewer insecticide applications than either fungicide or herbicide applications (Table 3). Between crops, there were more fungicide and insecticide applications in cranberry compared to blueberry, but the number of herbicide applications did not differ significantly among crops (Table 3). There was only a weak tendency toward field/bog size determining the number of pesticide applications (*p* = 0.062). The number of grower applications relative to the total number of residues detected in trapped pollen for each pesticide class (proportion pesticides applied) showed a similar pattern to the number of grower applications, a significant crop x pesticide class interaction. Table 3 depicts this pattern with the proportion of cranberry fungicides and insecticides, not significantly different from one another, but higher in proportion to the herbicide applications. The blueberry proportion applications followed the same pattern. There was no evidence for an effect of field/bog size on the proportions of pesticide use applications (*p* = 0.61).

The proportion of pesticide use class concentrations detected relative to the total residue concentration detected was not determined by field/bog size (*p* = 0.898) and demonstrated the following pattern. There were no significant differences among the three pesticide class concentration proportions in cranberry (Table 3). Grower-applied fungicide concentrations relative to the total residue concentrations were significantly greater than herbicide and insecticide relative concentrations which were not different from one another (Table 3). Among blueberries and cranberry, relative fungicide and insecticide concentrations in cranberry were not different from blueberry fungicide relative concentrations (Table 3). Risk quotients did not differ by crop, pesticide use class, or by the interaction of these two fixed effects. However, field/bog size did determine pesticide use class risk quotients (*F*_(1,15.3)_ = 5.181, *p* = 0.038). The slope coefficient was positive (β = 0.002) meaning that risk quotients, on average, were higher in larger fields and bogs than smaller fields and bogs.

#### 3.3.4. Field Decay Rates of Pesticide Residues in Cranberry Bogs and Blueberry Fields

**Figure 5:**
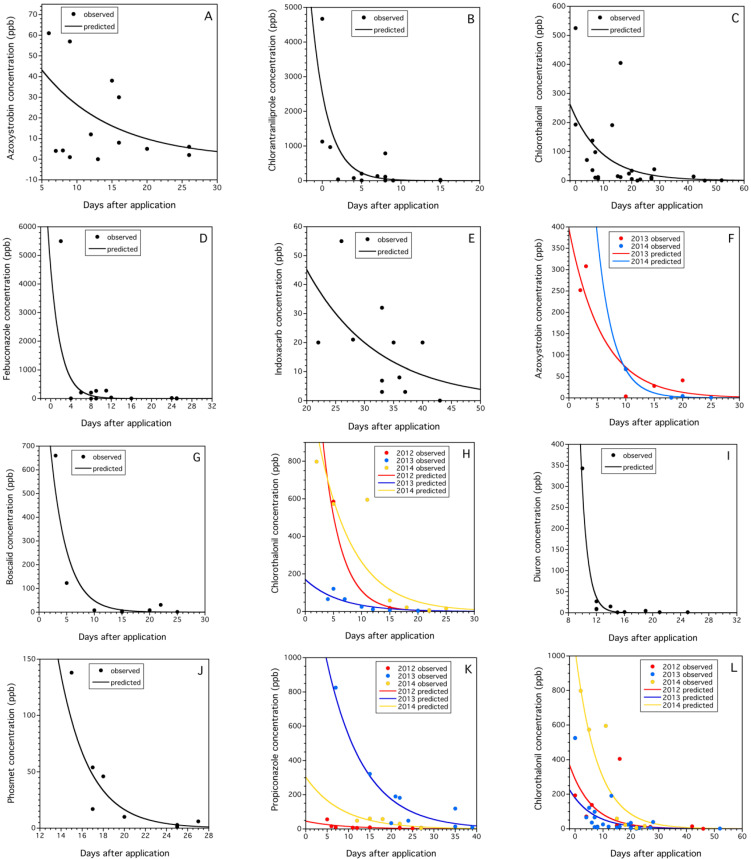
Pesticide residue concentrations (ppb) over time (days after application) modeled with a generalized linear model incorporating a Poisson distribution with a natural logarithm link function. Cranberry (**A**–**E**): azoxystrobin (**A**), chloratraniliprole (**B**), 4-hydroxy-chlorothalonil (**C**), fenbuconazole (**D**), indoxacarb (**E**); blueberry (**F**–**K**): azoxystrobin (**F**), boscalid (**G**), 4-hydroxy-chlorothalonil (**H**), diuron (**I**), phosmet (**J**), and propiconazole (**K**), and both crops, 4-hydroxy-chlorothalonil (**L**). Details of model fit and parameters are listed in Table 4. Half-lives or RT_50_s are listed in Table 5.

Quantification of field decay rates was possible for five pesticide residues associated with trapped honey bee pollen in cranberry bogs and six pesticide residues associated with trapped pollen in blueberry fields exhibited decay rates as a function of the days after application modeled by the Poisson distribution (Table 4). All models contained a significant days after application term that represents the exponential decay rate of the pesticide residues in the field after application (ranging across the crop systems and pesticides from 0.082 to 1.146). Three of the models contained a significant year term (fenbuconazole in cranberry, 4-hydroxy-chlorothalonil in blueberry, and propiconazole in blueberry), suggesting that residue rates of decay were similar between years, but at different levels of concentration across days after application between years. Only one model contained a significant year by days after application interaction term (azoxystrobin in blueberry, Figure 5F). This suggests that the decay rate varied by year for azoxystrobin, a result that we thought would be the case for most of the residues modeled. Figure 5A–L depicts the rapid rates of decay for the modeled pesticide residues in the field. The propiconazole daily rate of decay in this study in blueberry, −0.115 ± 0.017 (Figure 5K), was similar, only 41.9% and 2.6% higher than the daily rates of propiconazole decay on leaves and flowers during bloom estimated in 2010 (−0.081 ± 0.002) and 2011 (−0.112 ± 0.003) in blueberry fields, respectively [46]. On the other hand, the phosmet daily decay rate in trapped pollen that we estimated (−0.401 ± 0.064) was quite different, 179.7% and 1076.4%, higher than the daily rates of phosmet decay on fruit after bloom estimated in 2010 (−0.144 ± 0.024) and 2011 (−0.034 ± 0.016) in blueberry fields, respectively (Drummond, unpublished data). These two comparisons for phosmet suggest that the decay rates that we estimated for exposed pollen brought back to hives by honey bees may be similar to residue decay rates on leaves and flowers during bloom as observed with propiconazole, but possibly not for fruit just prior to harvest later in the growing season (late summer).

**Table 4:** Pesticide residue concentration (ppb) decay ^1^ over time (days) from date of application.

Crop	Pesticide ^1,2^	Predictors ^3,4^	Coefficients ^5^	*p* Value	Proportion Variance Explained ^6^	Mean Concentration ^7^ (ppb ± s.e.)
Cranberry	azoxystrobin	Intercept	3.977 ± 0.154	<0.0001	0.327	16.4 ± 5.7
		Days 4	−0.090 ± 0.012	<0.0001		
	chlorantrani-liprole	Intercept	8.172 ± 0.0155	<0.0001	0.841	481.4 ± 276.4
		Days	−0.484 ± 0.005	0.001		
	chlorothalonil	Intercept	5.388 ± 0.392	<0.0001	0.289	74.5 ± 26.3
		Days	−0.089 ± 0.037	0.016		
	fenbuconazole ^2^	Intercept	9.595 ± 0.185	<0.0001	0.907	467.9 ± 388.0
		Days	−0.525 ± 0.074	<0.0001		
	indoxacarb ^2^	Intercept	5.443 ± 1.215	<0.0001	0.267	17.2 ± 4.8
		Days	−0.082 ± 0.038	0.031		
Blueberry	azoxystrobin ^2^	Intercept	7.684 ± 0.699	<0.0001	0.899	78.3 ± 39.1
		Year	0.733 ± 0.669	0.273		
		Days	−0.349 ± 0.070	<0.0001		
		Year x Days	0.178 ± 0.089	0.046		
	boscalid	Intercept	7.235 ± 0.552	<0.0001	0.896	104.7 ± 80.6
		Days	−0.335 ± 0.093	0.004		
	4-hydroxy- chlorothalonil	Intercept	5.836 ± 0.629	<0.0001	0.837	133.4 ± 57.9
		Year (2012–14)	−1.609 ± 0.398	<0.0001		
		Year (2013–14)	−2.458 ± 0.423	<0.0001		
		Days	−0.159 ± 0.030	<0.0001		
	diuron ^2^	Intercept	16.151 ± 2.139	<0.0001	0.978	41.4 ± 33.6
		Days	−1.046 ± 0.206	<0.0001		
	phosmet	Intercept	10.769 ± 1.065	<0.0001	0.888	30.8 ± 13.9
		Days	−0.401 ± 0.064	<0.0001		
	propiconazole	Intercept	5.641 ± 0.355	<0.0001	0.945	80.5 ± 33.2
		Year (2012–14)	−1.828 ± 0.348	<0.0001		
		Year (2013–14)	1.816 ± 0.226	<0.0001		
		Days	−0.115 ± 0.017	<0.0001		
Both crops	4-hydroxy-chlorothalonil	Intercept	7.667 ± 0.419	<0.0001	0.603	89.4 ± 21.3
		Crop (cran-blue)	−0.724 ± 0.371	0.051		
		Year (2012–14)	−1.853 ± 0.561	0.001		
		Year (2013–14)	−2.147 ± 0.295	<0.0001		
		Days	−0.125 ± 0.028	<0.0001		

^1^ Poisson-generalized linear regression with a natural logarithm link function was used to model the data with the fixed effects year, days after application, and year x days after application, predictive variable selection was based upon the adaptive lasso technique. The parameters are derived from an additive exponential model. ^2^ Model is highly leveraged by one or two data points and when removed the model is no longer significant and so conclusions should be made with caution. ^3^ Predictors presented are those in the final model and significant at α = 0.05 or part of a significant interaction. ^4^ Days after application from when the trapped pollen was collected at the hives, the coefficient for days is the decay rate over time in days. ^5^ Predictive equation containing coefficients is exponentiated for estimating concentration for a given day after application. ^6^ Nagelkerke likelihood *r*^2^. ^7^ The mean concentration which was detected in trapped pollen over the fields sampled, concentrations are depicted in Figure 5.

Table 4 also shows that many of the decay rate models (8 of 11, Figure 5B,D,F–K) had high coefficient of determination rates (*r*^2^ > 0.80). This was surprising due to the mix of plant species pollen often found in pollen trap samples [47] and the different weather conditions that would have been experienced throughout bloom in the two crops. Inverse predictions of days after application for overall mean concentrations of the 11 modeled pesticide residues in collected pollen were not different among crops (*F*_(1,9)_ = 0.039, *p* = 0.846), 12.5 ± 5.3 days (cranberry) vs. 11.5 ± 1.6 days (blueberry).

In addition to modeling individual pesticide residues within each crop, we also modeled 4-hydroxy-chlorothalonil residues across both crops and all years (two years in cranberry and three in blueberry). We were able to model main effects, as well as the interactions between the three factors (crop, year, and days after application) despite the unbalanced nature of the dataset. The final model was a significant predictor of chlorothalonil residues in the field incorporating both crops and years (Figure 5L, Table 4). The factors significant for predicting chlorothalonil residues were the main effects: days after application, year, and crop. The crops effect verified the different residue levels in pollen over time between blueberry and cranberry (χ_(1)_^2^ = 3.804, *p* = 0.050). Years were also different in pollen concentrations of chlorothalonil over time (χ_(2)_^2^ = 57.811, *p* < 0.0001). Tukey HSD multiple comparisons revealed that the year 2014 had significantly higher chlorothalonil residues in pollen than pollen in 2012 and 2013, which were not different from one another. The estimation of the RT_50_ or half-life (days) of the concentration after grower application for an average level of chlorothalonil across all three crops and years (93.4 ± 26.9 ppb) was 10.7 days (95% CI: 8.9–18.9 days). This prediction was quite similar to the individual crop-specific estimates for blueberry and cranberry (8.9 and 11.9, blueberry and cranberry, respectively, Table 4). The predictive 4-hydroxy-chlorothalonil model fit in Figure 5L did not include the crop effect (*p* = 0.051) as it was marginally significant, more complex and more difficult to easily show predictions.

We compared our model estimations of RT_50_ for each of the pesticide residues fit to a Poisson density function error term (Table 4). We found that of the 12 model RT_50_ estimates, 11 could be compared to published RT_50_ values for these same residues. We could not compare the herbicide diuron because no published studies report field-level RT_50_ estimates. When we compared the 11 model estimates of RT_50_, 8 were in agreement with published values (agreement is defined as model estimates within the range of reported published RT_50_ values (Table 5). Only three model-estimated RT_50_ values did not agree with the range of published values.

## 4. Discussion

Working in two native perennial berry crops, our study provides a descriptive account of the average pesticide applications before and during bloom (up to the point of pollen sampling) and presents comparative residue data and estimated risk for honey bees that forage for pollen in commercial fields or bogs. For each crop, we established a baseline for real-world exposure levels and documented the common combinations of exposures. Such data are essential for future studies to document pesticide impact on honey bees. Clearly, our work shows that foragers and other nestmates would be chronically exposed to a combination of pesticides, likely with simultaneous exposure to multiple pesticide-use classes. These findings may be key to future work aimed at reducing risk to bees.

The pesticides that were applied and detected most often in lowbush blueberry were fungicides (12 different pesticide residues detected). Fungicides were also the most commonly detected class of pesticide found in a highbush blueberry study [48], a study that included cranberry [4], as well as in many other residue studies [49,50,51]. Research focused on fungicide exposure and threat to bees has recently soared. In particular, chlorothalonil, one of the world’s most extensively applied compounds for fungus and mildew management, and one of the most commonly applied and detected compounds in our berry study, has been implicated as a negative factor in aspects of bee health, including pathogen and parasite loads [4,5,52,53].

More insecticides were applied and detected in cranberry than blueberry. However, for the most part, the compounds detected are considered to have low toxicity to honey bees. Compared to an earlier study [5], with monitoring performed in 2007, several organophosphate residues were found in various bee matrices. In the current study, owing to shifts in pesticide use patterns, a single organophosphate (diazinon) was applied/detected. Diazion residues were largely responsible for the elevated RQ in cranberry trapped pollen. At the outset of this study, the process was underway to shift away from diazinon to a series of other mode of action options. The most widely used insecticide, chlorantraniliprole, is a diamide that functions as a ryanodine receptor modulator. In cranberry, it is usually sprayed twice during bloom to manage the native key insect pest, cranberry fruitworm (*Acrobasis vaccinii* Riley). Thus, sample collection events could occur near to the application explaining the high average residue (505.9 ± ppb) in pollen. It is likely that pollen is contaminated while foragers move through the vines and groom the body while packing the collected pollen. In contrast, indoxacarb (an oxadiazine) was used to manage cranberry weevil (*Anthonomus musculus* Say), and was applied well before bloom (average days between application and pollen sampling = 33.3), so residues persisted for weeks as flowers developed on vines. However, this insecticide exhibits differential toxicity based on route of exposure and is considered practically non-toxic when ingested [54]. In contrast, several of the members of the neonicotinoid class of compounds contribute to a highly toxic oral dose (imidacloprid, thiamethoxam, clothianidin) in other work; when analyses are undertaken at both local and area-wide scales, emergence of neonicotinoids are increasingly responsible for insecticide hazard. In our study, they contributed little to risk in cranberry and a low level in blueberry where acetamiprid and imidacloprid were applied and detected (only 7.7% of the blueberry fields had either acetamiprid or imidacloprid applied over the three-year study duration).

For the most frequently occurring pesticide and metabolite residues, low concentrations of three herbicides were detected in blueberry while in cranberry, high concentrations of a single herbicide, dichlorobenzamid, were commonly (proportion of samples: 0.88) detected with high average residues (350.8 ppb). The residues of dichlorobenzamid were a surprising finding since the herbicide is applied when the vines are dormant or after harvest. The compound must be remarkably long-lived to move into the developing flowers months after application. Glyphosate, one of the most widely used herbicides worldwide, was often detected in other surveys of bee-associated samples [55]. Glyphosate residues were not assessed in our study.

The honey bee colonies employed at our study sites were almost all migratory, and have been moved from other pollinating contracts in southern or western U.S. [56]. Pollen samples from both our crop systems typically had high residues of amitraz metabolites (proportion of samples: 0.63–0.72). However, blueberry had far higher varroacide residues compared to cranberry. Varroacides are often applied to colonies before or during their stint in Maine, and following blueberry pollination, these migratory colonies are moved to the later-blooming cranberry bogs in Massachusetts. Beekeepers do not appear to add more varroacide to the colonies since they were recently treated in blueberry. Under exposed and warm summer conditions in Massachusetts bogs, the rate of dissipation may be high. How field collected pollen becomes so contaminated likely occurs during pollen pellet processing as bees contaminated in the hive groom pollen grains from the body.

Insecticides were more commonly detected in cranberry pollen; the number of insecticide residues, residue concentration, and RQ were significantly higher in cranberry than blueberry. Comparing the crops, greater hazard may occur in cranberry where growers made a greater number (40% more) of pesticide applications and often treated during bloom. Further, pollen analysis across all pesticide classes indicated a higher concentration of residues and a greater risk to honey bees (based on RQ calculations) in cranberry. That being said, for both crops, pesticide RQ and combined sample RQ in this study were far below the EPA level of concern for acute exposure (0.4). Steering attention to sublethal effects and interactive effects of compounds may be a more profitable path to assessing pesticide impacts on colony losses. Even sublethal exposure of pesticides can lead to negative effects on colonies, such as reduced foraging efficiency, queen loss, pathogen infections, and compromised brood development [11].

A valuable contribution of our work is that we combined residue data with reliable reports from growers regarding their pesticide application dates. This allowed estimates of field decay rates, which can be a key decision factor for both regulators and growers to adjust timing and select pesticides to minimize hazardous pesticide exposure. Factors such as crop type and environmental conditions may influence pesticide persistence in pollen [57] and we saw both of these effects. Taken together, replication is required [58].

Using grower application records, we were able to determine that the highest concentration and most commonly identified pesticides were consistent with the grower reports. Further, we found that the majority of pesticides that were applied were detected in pollen (76.9% in blueberry and 66.7% in cranberry). Many Maine blueberry fields are embedded in Acadian or Boreal Forest landscapes, so bee foraging outside of lowbush blueberry fields in adjacent floral landscapes may not be common [59]. Cranberry beds are within the coastal sand plain region of MA, which is striking in its lack of diverse floral resources, but are embedded in the east coast urban corridor. Other than the mass blooming berry crops, extensive alternative foraging sites may be sparce. Large aggregations of contiguous lowbush blueberry fields in Maine and cranberry bogs in Massachusetts do exist and honey bee colonies placed in fields or bogs with neighboring adjacent blueberry fields may forage out of their immediate “home” field. Our data suggest that as field or bog size increases, the proportion of residues detected in trapped pollen that was applied by the grower increases. This might be expected as honey bees are more likely to forage inside of a bog or field that they are located in as the area of the site increases. This “trespass” foraging would be expected to commence both earlier and last for longer periods of time if the hive placement is in a small field or bog. [60] examined residues in samples of highbush blueberry flowers (*Vaccinium corymbosum* L.) and honey bee trapped pollen in Michigan (USA). They found that residues found for flowers were not similar to the residues found in trapped pollen, and concluded that most of the pesticide risk to honey bees was due to “off-farm” sources, and indicate that the colonies that were assessed were in regions of diverse crop production. In a New York apple study, [49] found that pesticide risk was mainly related to non-focal crop pollen, with over 60% of risk attributed to sources other than pesticide sprayed during the apple bloom period. Generally, honey bees may forage within a radius of several kilometers from the hive [61,62] so our similar finding of a substantial number of “off-farm” residues is not surprising and a likely source is other nearby blueberry crops. This is less likely for cranberry, since nearly half of the pesticides detected are not labelled for cranberry, and if bees are foraging at suburban sites, such as vector-treatment areas, golf courses, private landscapes, or nurseries, the source of pesticide exposure is more difficult to identify.

The fact that there is a significant gap in time since our study was conducted may suggest that the data may not fully reflect the current pesticide situation in these crops. However, our review of standard practices in commercial farms showed that there has been minimal shift in the pesticide landscape for both crops.

Based on RQ analysis, our pollen analysis results provide little evidence of lethal exposure risk or that lowbush blueberry and cranberry are high risk crops. However, looking ahead, we provide real-world exposure data and examples of pesticide class combinations that may inform future studies. In light of beekeeper reports of dead adults or malformed brood occurring around almond bloom, a laboratory bioassay investigated the effects of pesticides that are most commonly applied during orchard bloom. Few treated larvae survived a treatment combination of an SBI fungicide and chlorantraniliprole, dosed at the maximum label rate [63,64]. Following a long-term study relating pesticide effects to colony health, Traynor et al., 2021 [11] concluded that “myriad residues detected may result in poorly understood interaction effects” and emphasize the importance of sublethal/low risk exposure combined with other sources of stress.

## 5. Conclusions

We have demonstrated that honey bees brought in to pollinate native blueberry and cranberry crops in New England are at relatively low exposure risk to pesticide residues. Fungicides are the predominant residues in blueberry and insecticides are the dominant residues in cranberry. In both crops, grower-applied pesticides appeared to be the main source of risk to honey bees, but sources outside the field or bog that hives were placed also contributed. It is important that the main source of exposure is one that can be controlled by the growers. Providing field and local climate-realistic decay rates of the more commonly applied pesticides provides growers with a window of time that may allow them to control pests but also minimize exposure of honey bees to pesticide residues.

## Figures and Tables

**Table 1:** Most frequently occurring pesticide and metabolite residues and their concentrations in trapped pollen from hives in Massachusetts cranberry bogs (2012–2013), and Maine blueberry fields (2012–2014).

Cranberry Residues *^,1,2^	Frequency ^3^	Mean ppb ^4^	Blueberry Residues *^,1,2^	Frequency ^5^	Mean ppb ^4^
4-hydroxy- chlorothalonil ^F^	0.90	61.1 (17.4)	propiconazole ^F^	0.82	56.8 (22.7)
dichlorobenzamid ^H^	0.88	350.8 (104.2)	Amitraz metabolite DMPF *^,M^	0.72	811.6 (632.8)
chlorantraniliprole^I^	0.65	505.9 (260.2)	Amit-met DMPMF *^,M^ (methomyl coelutes)	0.67	226.0 (79.3)
Amit-met DMPMF *^,M^ (methomyl coelutes)	0.63	43.2 (43.2)	hexazinone ^H^	0.62	2.1 (1.1)
Amitraz metabolite DMPF *^,M^	0.63	99.1 (48.5)	4-hydroxy- chlorothalonil ^F^	0.54	61.7 (28.5)
azoxystrobin ^F^	0.58	10.7 (4.6)	atrazine ^H^	0.31	0.13 (0.1)
diazinon ^I^	0.55	8.4 (4.0)	carbendazim ^F^	0.28	0.6 (0.3)
fenbuconazole ^F^	0.50	165.2 (137.2)	diuron ^H^	0.26	10.6 (8.8)
indoxacarb ^I^	0.30	6.2 (1.9)	phosmet ^I^	0.23	24.9 (18.0)
methoxyfenozide ^I^	0.25	22.5 (16.7)	azoxystrobin ^F^	0.23	18.1 (10.1)
			boscalid ^F^	0.23	21.5 (17.1)
			pyraclostrobin *^,F^	0.21	3.4 (2.6)

* Denotes grower spray records do not indicate residue from sampled field. ^1^ Pesticide residue type—fungicide: ^F^, herbicide: ^H^, insecticide: ^I^, and miticide: ^M^. ^2^ Underlined residue compounds are shared frequently-occurring compounds between both cropping systems. ^3^ The proportion of samples that the residue compound was detected in 36 samples. ^4^ Number in parentheses is the standard error. ^5^ The proportion of samples that the residue compound was detected in 39 samples.

**Table 2:** Mixed models ^1^ constructed to determine relationships in total pesticides applied by growers to fields/bogs where colonies resided, and resulting residue concentrations, and risk quotients as a proportion of total residues detected in trapped pollen. Pesticide applications were derived from grower pesticide spray records and assumed beekeeper application of varroacides to the colonies. Treatment comparisons were made among crops.

Test Description	Dependent Variable	F-Statistic ^2^	*p*-Value	Treatment Means
grower total applications	total pesticide applications ^3^/year/farm	crop: *F*_(1,42.8)_ = 10.045	*p* = 0.003	cranberry = 6.3 ± 0.7 blueberry = 3.7 ± 0.4
	proportion pesticide applications ^4^/year/farm	crop: *F*_(1,53.0)_ = 14.459	*p* = 0.0004	cranberry = 0.87 ± 0.06 blueberry = 0.54 ± 0.05
	proportion pesticide applications ^5^ detected/year/farm	crop: *F*_(1,43.1)_ = 0.036	*p* = 0.851	cranberry = 0.50 ± 0.09 blueberry = 0.52 ± 0.05
	proportion pesticide applications ^6^ detected/year/farm	crop: *F*_(1,45.0)_ = 6.906	*p* = 0.012	cranberry = 0.55 ± 0.08 blueberry = 0.90 ± 0.04
	proportion residue concentrations ^7^/year/farm	crop: *F*_(1,40.9)_ = 7.566	*p* = 0.008	cranberry = 0.61 ± 0.09 blueberry = 0.40 ± 0.07
	proportion residue RQ ^8^/year/farm	crop: *F*_(1,36.7)_ = 0.123	*p* = 0.728	cranberry = 0.35 ± 0.11 blueberry = 0.40 ± 0.05
beekeeper applied varroacides	proportion varroacide compounds ^9^ applied by beekeepers/year/farm	crop *F*_(1,46.1)_ = 19.972	*p* < 0.0001	cranberry = 0.02 ± 0.06 blueberry = 0.27 ± 0.05
	proportion of varroacide concentrations ^7^/year/farm	crop *F*_(1,51.0)_ = 18.421	*p* < 0.0001	cranberry = 0.02 ± 0.05 blueberry = 0.39 ± 0.03
	proportion of varroacide RQ ^8^/year/farm	crop *F*_(1,51.0)_ = 2.577	*p* = 0.115	cranberry = 0.16 ± 0.97 blueberry = 0.33 ± 0.04

^1^ Mixed models include fixed effects of crop, and the covariate field or bog size (ha) and its interaction with crop, random effects were year and farm. ^2^ Based upon corrected degrees of freedom for mixed model fixed effects, Satterthwaite’s method. ^3^ Pesticides applied per year, where year refers to applications up to and including bloom, but not after pollination. ^4^ Number of applied pesticides relative to total number of unique pesticide residues detected in trapped pollen. ^5^ Number of applied pesticides that were also detected as residues in trapped pollen relative to the total number of unique pesticide residues detected in the same trapped pollen sample. ^6^ Number of applied pesticides that were detected as residues in trapped pollen relative to the total number of pesticide residues detected that matched the pesticides applied by growers in the same trapped pollen sample. ^7^ Concentrations of residues matched with the grower-applied pesticides relative to the total residue concentration detected in trapped pollen. ^8^ Risk quotients (RQ) of residues matched with the grower-applied pesticides relative to the total residue risk quotients detected in trapped pollen. ^9^ Number of assumed varroacide residues detected in trapped pollen relative to the total number of unique residue compounds detected.

**Table 3:** Mixed models ^1^ constructed to determine relationships in specific pesticides use classes (fungicides, herbicides, and insecticides) applied by growers to fields/bogs where colonies resided, and resulting residue concentrations, and risk quotients as a proportion of total residues detected in trapped pollen. Pesticide applications were derived from grower pesticide spray records. Treatment comparisons were made between crops, pesticide use classes, and the interaction between these two fixed treatment independent categorical variables.

Dependent Variable	F-Statistic ^2^	*p*-Value	Blueberry Treatment Means	Cranberry Treatment Means
number pesticide use class applications ^3^/year/farm	crop:		fungicide:	fungicide:
	*F*_(1,41.6)_ = 24.130	*p* < 0.0001	1.91 ± 0.21 ^bc^	3.53 ± 0.34 ^a^
	pesticide use class:		herbicide:	herbicide:
	*F*_(2,118.8)_ = 17.161	*p* < 0.0001	1.35 ± 0.21 ^c^	1.58 ± 0.34 ^c^
	interaction:		insecticide:	insecticide:
	*F*_(2,118.8)_ = 13.920	*p* < 0.0001	0.35 ± 0.21 ^d^	2.99 ± 0.34 ^ab^
proportion pesticide use class applications ^4^/year/farm	crop:		fungicide:	fungicide:
	*F*_(1,34.0)_ = 19.091	*p* < 0.0001	0.27 ± 0.03 ^bc^	0.50 ± 0.06 ^a^
	pesticide use class:		herbicide:	herbicide:
	*F*_(2,118.8)_ = 10.744	*p* < 0.0001	0.21 ± 0.03 ^c^	0.25 ± 0.06 ^bc^
	interaction:		insecticide:	insecticide:
	*F*_(2,118.9)_ = 8.979	*p* < 0.0001	0.04 ± 0.03 ^d^	0.41 ± 0.06 ^ab^
proportion pesticide use class residue con-centration ^5^/year/farm	crop:		fungicide:	fungicide:
	*F*_(1,161.0)_ = 1.989	*p* = 0.160	0.27 ± 0.03 ^a^	0.13 ± 0.05 ^abc^
	pesticide use class:		herbicide:	herbicide:
	*F*_(2,161.0)_ = 2.706	*p* = 0.069	0.09 ± 0.03 ^c^	0.09 ± 0.05 ^bc^
	interaction:		insecticide:	insecticide:
	*F*(2,161.0) = 9.216	*p* = 0.0002	0.03 ± 0.03 ^c^	0.31 ± 0.05 ^ab^
proportion pesticide use class risk quotients ^6^/year/farm	crop:		fungicide:	fungicide:
	*F*_(1,16.8)_ = 3.578	*p* = 0.076	0.18 ± 0.3	0.08 ± 0.05
	pesticide use class:		herbicide:	herbicide:
	*F*_(2,123.6)_ = 2.062	*p* = 0.132	0.66 ± 0.03	0.024 ± 0.05
	interaction:		insecticide:	insecticide:
	*F*_(2,123.6)_ = 0.142	*p* = 0.867	0.17 ± 0.03	0.10 ± 0.05

^1^ Mixed models include fixed effects of crop, pesticide use class, the crop by pesticide use class interaction, and the covariate field or bog size (ha) and its interaction with crop, random effects were year and farm. ^2^ Based upon corrected degrees of freedom for mixed model fixed effects, Satterthwaite’s method. ^3^ Use class pesticides applied per year, where year refers to applications up to and including bloom, but not after pollination. ^4^ Number of applied use class pesticides relative to total number of unique pesticide residues detected in trapped pollen. ^5^ Concentrations of use class pesticide residues matched with the grower-applied pesticides relative to the total residue concentration detected in trapped pollen. ^6^ RQ of use class pesticide residues matched with the grower-applied pesticides relative to the total residue RQ in trapped pollen. Treatments with the same letters are not significantly different from one another.

**Table 5:** Pesticide residue concentration RT_50_ (half-life) in days from date of grower application.

Crop	Pesticide Residue Model ^1^	Model Predicted RT_50_ (Days) ^2^	Published Mean and Range RT_50_ (Days) ^3^	Number of Published Studies ^4^	Model Agreement with Published ^5^
Cranberry	azoxystrobin	13.0 (11.6–14.7)	8.0 (0.4–17.5)	17	yes
	chlorantrani-liprole	3.5 (1.9–7.8)	4.3 (2.2–9.9)	7	yes
	4-hydroxy-chlorothalonil	11.9 (5.5–36.9)	5.0 (1.7–12.7)	21	yes
	fenbuconazole	2.2 (−2.0–6.3)	10.2 (5.7–14.8)	2	no
	indoxacarb	31.8 (19.9–45.7)	1.6 (0.8–2.4)	2	no
Blueberry	azoxystrobin	9.5 (7.8–15.4)	8.0 (0.4–17.5)	17	yes
	boscalid	7.7 (5.9–15.6)	5.5 (1.3–11)	8	yes
	4-hydroxy-chlorothalonil	8.9 (6.4–12.3)	5.0 (1.7–12.7)	21	yes
	diuron	11.9 (11.4–12.9)	na	0	no published data
	phosmet	18.3 (17.5–19.5)	2.7 (1.6–4.6)	5	no
	propiconazole	12.5 (14.6–16.2)	7.4 (1.0–16.9)	10	yes
Both crops	4-hydroxy-chlorothalonil	10.2 (8.5–16.3)	5.0 (1.7–12.7)	21	yes

^1^ All models presented are those with all data points used despite some models being leveraged by 1 or 2 data points. ^2^ Model-estimated RT_50_ along with 95% confidence intervals in parentheses. ^3^ Published mean RT_50_ along with the range of published RT_50_ values in parentheses. The data for the published RT_50_ values were extracted from the IUPAC Pesticide Properties Database (PPDB), see Methods for URL. ^4^ Number of published studies that were used to estimate the mean and range of RT_50_ values for each pesticide residue. ^5^ A conclusion of agreement (yes) when the model-estimated RT_50_ was within the minimum to maximum range of published RT_50_ values. If the model-estimated RT_50_ was outside the range, then the conclusion was that the model estimate did not agree (no), with the published range of RT_50_ values.

## Data Availability

Data will be shared upon request.

## Appendix A

In order to visualize the residue mixture across both crops, ordination (Principal Component Analysis) across many measures of residues detected in trapped pollen was used. Using all of the residue measures (# detections, concentration (ppb), risk (RQ), and field size, it can be seen that while pesticide exposure to honey bees is different between Maine blueberry and Massachusetts cranberry, there is overlap between the two-dimensional (principal components axes 1 and 2) pesticide residue space (Figure A1A). Both eigenvalues (first two principal components) are significant and explain 62.2% of the variation in pesticide residues detected in trapped honey bee pollen across both crops. Figure A1B shows a biplot of the orthogonal factor loadings. The factor plot shows that miticide residues used for management of *Varroa* mites are a main determinant of the blueberry residue multi-dimensional population, whereas, insecticide residues are a determinant of cranberry residue multi-dimensional population.
